# The Health Hazards of Volcanoes: First Evidence of Neuroinflammation in the Hippocampus of Mice Exposed to Active Volcanic Surroundings

**DOI:** 10.1155/2021/5891095

**Published:** 2021-10-11

**Authors:** A. Navarro-Sempere, P. Martínez-Peinado, A. S. Rodrigues, P. V. Garcia, R. Camarinho, M. García, Y. Segovia

**Affiliations:** ^1^Department of Biotechnology, Faculty of Science, University of Alicante, Apartado 99, 03080 Alicante, Spain; ^2^Faculty of Sciences and Technology, University of the Azores, 9501-801 Ponta Delgada, Portugal; ^3^IVAR, Research Institute for Volcanology and Risk Assessment, University of the Azores, 9501-801 Ponta Delgada, Portugal; ^4^CE3c, Centre for Ecology, Evolution and Environmental Changes and Azorean Biodiversity Group, University of the Azores, 9501-801 Ponta Delgada, Portugal

## Abstract

Neuroinflammation is a process related to the onset of neurodegenerative diseases; one of the hallmarks of this process is microglial reactivation and the secretion by these cells of proinflammatory cytokines such as TNF*α*. Numerous studies report the relationship between neuroinflammatory processes and exposure to anthropogenic air pollutants, but few refer to natural pollutants. Volcanoes are highly inhabited natural sources of environmental pollution that induce changes in the nervous system, such as reactive astrogliosis or the blood-brain barrier breakdown in exposed individuals; however, no neuroinflammatory event has been yet defined. To this purpose, we studied resting microglia, reactive microglia, and TNF*α* production in the brains of mice chronically exposed to an active volcanic environment on the island of São Miguel (Azores, Portugal). For the first time, we demonstrate a proliferation of microglial cells and an increase in reactive microglia, as well an increase in TNF*α* secretion, in the central nervous system of individuals exposed to volcanogenic pollutants.

## 1. Introduction

The role of microglial cells in neuroinflammatory events currently represents one of the main research areas in neurobiology due to the potential therapeutic application. Microglia is a population of resident immune cells in the central nervous system (CNS) being a front-line defence against a threat to the nervous tissue [1, 2]. Although these cells are present throughout the nervous system, they predominate in the grey matter [3], being abundant in areas such as the hippocampus, olfactory telencephalon, basal ganglia, and the substantia nigra [4]. Normally, in the mature health brain, microglial cells are found in their resting form, exhibiting a rounded cell body, which generally remains fixed, and long and highly branched prolongations. These ramifications undergo cycles of formation and retraction that give the cells pronounced motility, thus enabling the monitoring of the cellular neighbourhood [5], safeguarding the homeostasis of the nervous system, and clearing the parenchyma of accumulated metabolic products and debris from deteriorated tissues. In addition, microglial cells show another peculiarity: upon an immune stimulus or CNS damage, they are rapidly activated undergoing a dramatic morphological transformation and exhibiting a set of surface molecules [6–8] including CD68, a transmembrane protein on both lysosomal [9] and plasma membrane [10] which is present on monocytes and macrophages, acting as a modulator of the immune response [8]. Furthermore, in response to damage, reactive microglia secrete a wide range of trophic factors and cytokines that can act in either beneficial or detrimental ways on the surrounding cells [11–15]. Microglia activation is the hallmark of neuroinflammation.

Multiple neuroinflammatory processes are regulated by these cytokines [16]. Tumour necrosis factor alpha (TNF*α*), one of the best characterised proinflammatory cytokines, plays both a homeostatic and pathologic role in the CNS [17]. In the healthy nervous system, TNF*α* is involved in processes such as synaptic plasticity [18, 19] or learning and memory [20, 21]. However, in face of damage or threat to the nervous system, some glial cells, mostly astrocytes and microglia, and certain neuronal populations can produce this cytokine in large quantities. This released is considered a key component of neuroinflammation [22] that leads to a wide range of double-edged sword responses: it has a protective role at acute levels but can contribute to tissue damage and trigger disease when it is a sustained response over time [23]. There are several studies in the literature linking chronic neuroinflammation to neuronal death [23–27] and thus to neurodegenerative diseases such as Alzheimer's disease [28–34], Parkinson's disease [35–37], or multiple sclerosis [38–41].

The occurrence of neuroinflammatory processes as well as neurodegenerative diseases in relation to chronic exposure to environmental pollution has been extensively studied [42–52]. However, all the literature refers to anthropogenic pollution, and little is known about the effect of natural pollution on health, even though there are large natural sources of pollution, such as volcanoes, which can cause health problems.

Volcanoes are attractive for human settlements due to the fertility of their soils and their touristic interest [53–55], but they are also dangerous due to the geochemical processes that take place during both eruptive and noneruptive periods. Considering that volcanoes are a major source of natural pollution, with emissions of certain gases comparable to anthropogenic emissions [56], and that an estimated 44 million people live within 10 km of an active volcano [57], it is very interesting to study the effect of such exposure on health.

The island of São Miguel (Azores archipelago, Portugal) has three active volcanoes: Sete Cidades, Fogo, and Furnas. The latter, considered one of the most active in the archipelago due to its very marked volcanic activity, exhibits different hydrothermal manifestations such as strong ground degassing, thermal and cold CO_2_ springs, and fumarolic fields. Although it is estimated that Furnas volcano emits 1000 tons of CO_2_ per day [58], the village of Furnas, with about 1700 inhabitants, is located inside the volcano crater. Numerous studies have shown that people chronically exposed to such volcanic manifestations can develop chronic bronchitis and other respiratory diseases [55, 59] and certain types of cancer such as lip, oral cavity, or pharyngeal cancer [60]. However, the respiratory system is not the only one that reacts to exposure to such a hostile environment; changes in the CNS have already been reported, such as the accumulation of inorganic mercury in different areas of the brain parenchyma [61], which suggests a breakdown in the blood-brain barrier, as well as astrocyte reactivity and dysfunction in important areas of the brain such as the hippocampus [62].

Since, as mentioned above, the literature focuses on neuroinflammation as one of the main events following long-term exposure to air pollutants and as a trigger for future neurodegenerative diseases, our work is aimed at detecting a neuroinflammatory response in individuals chronically exposed to volcanic pollutants by studying microglia (resting and reactive form) and the proinflammatory cytokine TNF*α*.

## 2. Material and Methods

### 2.1. Study Areas and Animal Capture

Two groups of feral mice, *Mus musculus*, were captured alive in two different areas of the island of São Miguel: the village of Furnas and Rabo de Peixe. The Furnas village, built on the degassing soil of the crater of the homonymous volcano, presents important manifestations of volcanic activity such as soil degassing. This phenomenon is responsible for the release of hazardous gases such as radon (^222^Rn), hydrogen sulphide (H_2_S), and carbon dioxide (CO_2_) among others, as well as volatile metals into the atmosphere [63–65]. On the other hand, Rabo de Peixe village, used as a control site, is located 20 km from the exposed area and shows no evidence of active volcanism or sources of anthropogenic contamination. In addition, this area is placed near the coast, presenting a high air renewal rate.

The selection of *Mus musculus* as a surrogate species is due to several important reasons: on the one hand, the fact that it shares habitat with humans, being sometimes captured even inside inhabited houses, both in the volcanically active area and in the reference area. On the other hand, different authors have reported the robustness of research using feral specimens in the evaluation of the effects of contaminant exposure on individuals, compared to laboratory studies, since the latter may present discrepancies with reality in terms of diet, animal behaviour, and even the mixture of contaminants [66, 67].

*Mus musculus* individuals (Furnas, *N* = 5 and Rabo de Peixe, *N* = 5) were captured by trapping at different points in the study areas and transferred to the laboratory in the shortest possible time for processing. To avoid any animal distress, mice were anaesthetised with isofluorane until an optimal level of anaesthesia was reached and then transcardially perfused with phosphate buffered saline followed by 4% paraformaldehyde solution (PFA). After perfusion, the animals were necropsied by surgical extraction of the brain, which was fixed by immersion in 4% PFA overnight at 4°C. Sex, body weight, and age were recorded for each individual; the latter was estimated using the dry weight of the crystalline lens according to the methodology of Quere and Vincent [68]. Individuals weighing less than 10 g were discarded for this study.

Experimental procedures were approved by the Ethics Committee of the University of Azores (REF: 10/2020). All procedures were performed conformed with the recommendations of the European Convention for the Protection of Vertebrate Animals used for Experimental and Other Scientific Purposes (ETS 123), directive 2010/63EU and Portuguese law decree (DL 113/2013).

### 2.2. Tissue Processing and Immunofluorescence Assay

After overnight fixation in 4% PFA, the brains were processed for paraffin embedding, and once included, serial sagittal 4 *μ*m thickness sections were cut using a microtome (Microm HM 340E). The slides were dewaxed using xylol and hydrated in decreasing concentration of ethanol until PBS 0.1 M, and after hydration, the immunofluorescence assay was performed as follows. Briefly, brain sections containing the hippocampus were subjected to heat-induced epitope retrieval and blocked with 10% BSA for 90 minutes at room temperature. Then, samples were immunolabeled at 4°C overnight using the following primary antibodies at 1 : 100 dilution: anti-Iba1 (GTX101495, Genetex), anti-CD68 (GTX37743, Genetex), and anti-TNF*α* (GTX110520, Genetex). The next day, the slides were washed and incubated with the secondary antibody (SAB4600310, Sigma Aldrich Co.) diluted at 1 : 500 during 3 hours at room temperature. Then, sections were washed repeatedly and covered with Vectashield medium (Vector Laboratories, Burlingame CA) containing DAPI to counterstain nuclei.

### 2.3. Quantitative Analysis

Confocal images of the hippocampus were taken using Zeiss confocal scanning microscope (LSM 800), and the immunofluorescence assessment was carried out following the methodology reported by Navarro et al. [61]. Altogether, from each individual, three coronal sections every 150 *μ*m were taken and analysed keeping constant pinhole, contrast, and brightness. From each section, photographs were obtained at 20x magnification, every 0.5 *μ*m z-step and assembled in an orthogonal projection through the Zen Blue software.

**Figure 1:**
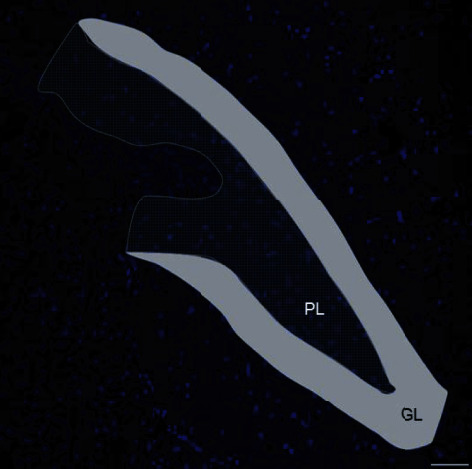
Region of interest (ROI) for the different analyses. The hippocampal dentate gyrus is divided in two areas: granular layer (GL) and polymorphic layer (PL). Scale bar: 50 *μ*m.

The region of interest (ROI) of our experiments was a specific hippocampal formation (Figure 1), the dentate gyrus. In this brain circumvolution, two subareas were analysed: the polymorphic layer (PL) and the granular layer (GL). The total number of immunopositive cells per *μ*m^2^ in both subareas was counted and expressed in cells/mm^2^ using the Image J software. For this count, three different researchers performed blindly these quantifications and the results were averaged.

### 2.4. Statistical Analysis

Data regarding the density of Iba1^+^ and CD68^+^ cells in mouse dentate gyrus from both study locations was compared using Student's *t*-test, and a *p* value of less than 0.05 was considered as statistically significant. The software Graph Pad Prism (Graph Pad Software Inc., La Jolla, CA, USA) was used to conduct all the statistical analysis.

## 3. Results

All samples used in this study correspond to male individuals. No statistical differences were found between both study groups in age (Furnas: 204 ± 9 days old and Rabo de Peixe: 213 ± 13 days old; *p* = 0.193, Student's *t*-test) and weight (Furnas: 13.55 ± 2.42 g and Rabo de Peixe: 14.10 ± 0.90 g; *p* = 0.728, Student's *t*-test).

### 3.1. Iba1 Expression Is Increased in Individuals Chronically Exposed to Volcanic Environments

Microglial cells were confirmed in this study by staining with the anti-Iba1 antibody (ionizing calcium-binding adaptor molecule 1) in the dentate gyrus from Furnas and Rabo de Peixe mice. It is a protein that consistently is expressed on all microglial subtypes.

**Figure 2:**
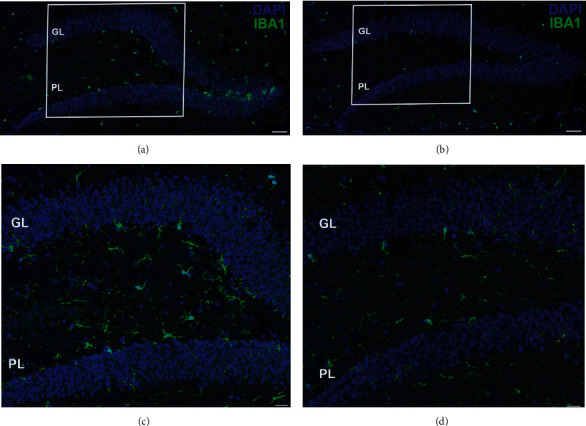
Expression of the microglial marker Iba1 in the dentate gyrus of mice from (a, b) Furnas and (c, d) Rabo de Peixe. Scale bar: 50 *μ*m. Magnification of a section of the total microglia in the dentate gyrus of (c) Furnas and (d) Rabo de Peixe. GL: granular layer; PL: polymorphic layer. Scale bar: 20 *μ*m.

Qualitative comparison from immunofluorescence analysis of Iba1 revealed that the staining pattern for Iba1 was much higher in samples from mice living in the Furnas region, both in the polymorphic layer and in the granular layer of the dentate gyrus (Figure 2).

**Figure 3:**
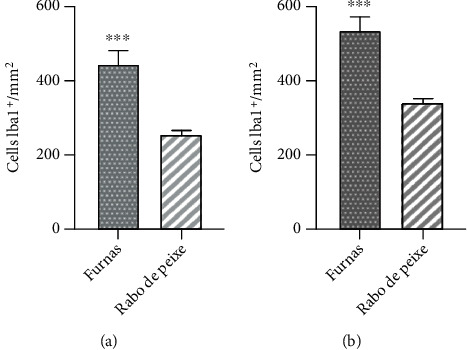
Quantification of Iba1^+^ cells/mm^2^ in both dentate gyrus layers: (a) granular and (b) polymorphic layers of mice from the two study locations. Data reported in the bar graph is represented as mean ± SEM. The statistical analysis was performed using Student's *t*-test, ^∗∗∗^*p* < 0.001.

The number of Iba1 positive cells in the immunofluorescence assay was quantified in each layer of the dentate gyrus: granular and polymorphic layer, from individuals captured in the two study areas. An increase in the number of these cells was observed in those animals chronically exposed to volcanogenic pollutants compared to individuals from Rabo de Peixe, in both the granular layer (448.71 ± 33.41 cells/mm^2^ vs. 258.45 ± 9.42 cells/mm^2^; ^∗∗∗^*p* > 0.001) and the polymorphic layer (535.67 ± 31.47 vs. 341.84 ± 13.08; ^∗∗∗^*p* < 0.001) (Figure 3).

### 3.2. Expression of CD68, a Marker of Active Microglia

CD68 (macrosialin in mice) is one of the most helpful and descriptive markers of microglial function. This protein is localised to the lysosomal membrane of microglia and is upregulated in active phagocytic cells [69]. It is, therefore, a marker of microglial activation with phagocytic activity.

**Figure 4:**
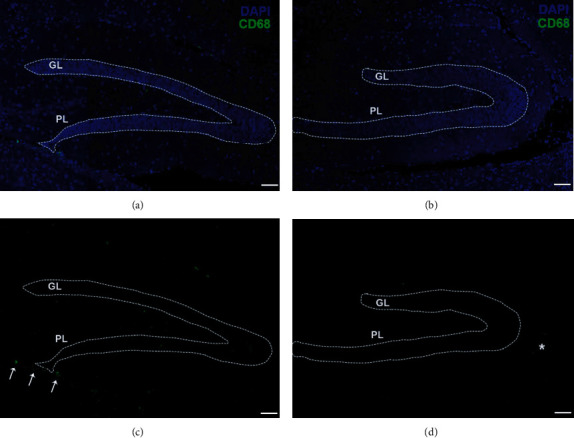
Analysis of active microglia in GL and PL of mice chronically exposed to (a, b) volcanogenic pollutants and from the (c, d) control site. CD68 (green) is expressed in both dentate gyrus layers only in those animals inhabiting (b) active volcanic environments; no immunofluorescence signal is observed in the dentate gyrus of mice from (d) Rabo de Peixe. Note a higher number of CD68^+^ cells in the surrounding area of the choroid plexus in Furnas' mice (white arrows) were compared to a few cells observed in the vicinity of the plexus in control site mice (asterisk). GL: granular layer; PL: polymorphic layer. Scale bar: 50 *μ*m.

Immunofluorescence evaluation of CD68^+^ positive cells in both layers of the hippocampal dentate gyrus revealed that chronic exposure to an active volcanic environment induces the increment of these cells in the assessed tissue (Figure 4). Likewise, an important CD68^+^ immunofluorescent staining was observed in the choroid plexus and the area surrounding it in individuals from Furnas village; such staining was less evident in rodents from Rabo de Peixe.

### 3.3. Immunofluorescence and Localisation of TNF*α* in the Dentate Gyrus of the Hippocampus of Exposed Mice

TNF*α* was used as a proinflammatory marker. TNF*α* expression was evaluated in mouse dentate gyrus cells from both locations by immunofluorescence staining. The immunoreactivity is localised in the intracellular spaces around the nucleus of neurons in both polymorphic and granular layers of the dentate gyrus of mice chronically exposed to a volcanic environment. In contrast, no reaction was detected in the perikaryon of the dentate gyrus neurons of the Rabo de Peixe samples.

**Figure 5:**
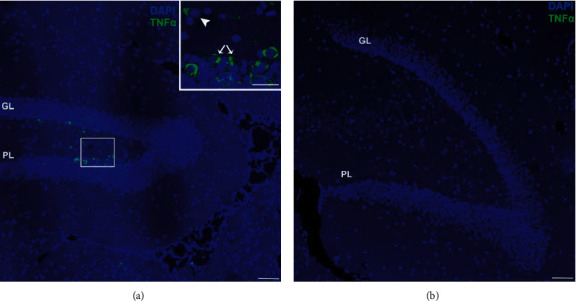
Immunofluorescence assay of TNF*α* in the dentate gyrus of rodents inhabiting (a) Furnas village and (b) Rabo de Peixe. Accumulation of TNF*α* is evident inside some cells located in the subgranular zone, compatible with neural stem cells (inset, white arrows) and in the hilum, compatible with mossy cells (inset, arrowhead). GL: granular layer; PL: polymorphic layer. Scale bar: 50 *μ*m.

In the samples from the animals inhabiting Furnas, the immunoreactive cells are found in the subgranular zone (SGZ) located on the inner surface of the granule cell layer. These cells could be compatible with neural stem cells (Figure 5). As for the polymorphic layer, in the image, we can observe a minor marking, consistent with the mossy cells of this layer.

## 4. Discussion

Air pollution is a major public health concern due to the large number of studies that have linked long-term exposure to various health effects. Although some studies have shown a link between chronic exposure to anthropogenic pollution and effects on the nervous system, only a few have focused on studying these effects regarding volcanic pollution [61, 62]. The Azores archipelago is a volcanic area with several manifestations of active volcanism, making it an ideal place to study environmental health problems [70]. Previous research has shown that volcanic areas are associated with an increased incidence of a wide range of diseases [55, 59, 60, 71–74]. However, very little is known about the consequences on the nervous system of people inhabiting volcanic environments.

Both volcanoes and geothermal areas are associated with emissions of a variety of gases that classically include carbon dioxide (CO_2_), sulphur dioxide (SO_2_), hydrogen chloride (HCl), hydrogen fluoride (HF), hydrogen sulphide (H_2_S), carbon monoxide (CO), radon (Rn), and some heavy metals such as lead and mercury, among others [56]. Therefore, volcanoes are considered an important source of pollutants, including air pollutants, that can damage the health of individuals living in these natural spaces.

Air pollution is a prevalent proinflammatory stimulus for the CNS, which until a few decades ago was not known to be involved as a risk factor for neurodegenerative diseases [45, 49]. For this reason, the rationale of this work has been to relate volcanic contamination to proinflammatory events in the CNS individuals chronically exposed to volcanic contamination. For this purpose, we have studied microglial cells.

As mentioned above, microglia are immunoregulatory cells that play an important role in the healthy and diseased CNS. They help maintain the homeostasis of the brain environment under normal conditions but show a strong reaction in response to adverse conditions, becoming activated microglia and adopting an amoeboid phenotype. These microglia proliferate and migrate to the site of injury or damage, where they perform a protective function, removing cellular debris [75–77]. On the other hand, overactivation of microglia, with excess production of inflammatory mediators, can have neurotoxic consequences. Whether microglial function in neurodegenerative diseases is beneficial but insufficient or whether microglia are only effective in the early stages of the disease but become detrimental in later stages is still unknown.

The intense reaction of microglia is collectively termed “microgliosis.” As revealed by Li and Zhang [78], this may exist at least three sources for microgliosis in the adult CNS: local proliferation of reactive microglia, infiltration of blood-derived cells, and mobilization of latent progenitors within the CNS. Each or all of these sources may play a role in microgliosis in different pathological conditions. Alterations in microglia functionality are therefore implicated in brain neurodegeneration.

Our results show several proinflammatory events in the dentate gyrus of animals chronically exposed to an active volcanic environment. These events are the proliferation of the microglial population, the presence of activated microglial cells with phagocytic activity, and intracellular accumulation of TNF*α*. The dentate gyrus is a very relevant area of the hippocampal formation, not only because it has been described as highly sensitive to oxidative stress [79], but because it is where the adult neurogenesis takes place [80].

Proinflammatory mediators produced in epithelial and olfactory tissue as a result of chronic exposure to volcanic pollutants can induce systemic inflammation and reach the brain parenchyma through the breakdown of the blood-brain barrier. This inflammation is accompanied by the production of different proinflammatory cytokines, such as IL1*β*, IL6, or TNF*α*, for which brain vessel endothelial cells exhibit constitutive and induced receptors. Endothelial cytokine-receptor binding activates endothelial cells thereby disrupting the blood-brain barrier. Our study focused on the proinflammatory cytokine TNF*α* revealed its increase in mice from Furnas. Camarinho et al. [71] also observed its overproduction in the respiratory tissue of mice living in the same location as our study (Furnas village). It is not unreasonable to think that this same cytokine could be present in the CNS from two sources: either by entry from the systemic circulation or by being produced in the CNS itself. Within the central nervous system, microglia, astrocytes, and neurons are major sources of TNF*α* [81–86]. In fact, we have detected immunoreactivity in cells located in the subgranular zone of the dentate gyrus in chronically exposed animals. These cells, whose location and size are compatible with neural stem cells (NSCs), must have received a proinflammatory signal of environmental origin that led them to activate the NFkb transcription machinery, which regulates numerous genes, including those coding for proinflammatory cytokines [87–90]. Therefore, the presence of cytokines in the extracellular milieu may be a stimulus for these cells to initiate TNF*α* production and thus enter a proinflammatory loop. It is important to note that this staining was not observed in individuals living in Rabo de Peixe, our control population.

As demonstrated by Widera et al. [91], following CNS injury, TNF*α* plays a critical role in the development of pathology and inflammation, as well as activating NSC proliferation, triggering a neuroprotective mechanism. In this regard, Pluchino et al. [92] demonstrated that during CNS inflammation, NSCs were able to secrete neuroprotective cytokines. Neuroinflammation may be beneficial as a tissue protector process; however, if this is sustained over the time can lead to a chronic neuroinflammation cycle essential for the pathogenesis and progression of neurodegenerative diseases, such as Alzheimer's disease, Parkinson's disease, Huntington's disease, and multiple sclerosis [93, 94]. In addition, a chronic neuroinflammation condition contributes to both cognitive impairment [95] and memory formation, disrupting the acquisition and impairing the consolidation/reconsolidation process [96, 97].

The proliferation of microglia, quantified by the marker Iba1, and its morphological change towards a phagocytic form, in which CD68 expression increases, as observed in the dentate gyrus of exposed animals compared to those captured in Rabo de Peixe, suggest that an innate immune response of the CNS to volcanic contamination is taking place. This type of response by microglial cells is consistent with that reported by numerous papers focusing on anthropogenic pollution [43, 45, 98–101]. Importantly, microglial activation is necessary to repair the injured microenvironment by removing cellular debris. However, as a consequence of this activation, these microglial cells can damage living neurons through their phagocytic capacity or by releasing cytokines [102].

Moreover, in addition to the existence of CD68^+^ cells in the hippocampal dentate gyrus, immunofluorescence has also been observed in the ventricles and areas adjacent to them. This finding indicates that systemic macrophage infiltration of the brain parenchyma may be occurring, preceded by a loss of BBB integrity. Again, it is important to note that, in mice captured in Rabo de Peixe, no immunofluorescence signal was observed in the vicinity of the ventricles. This agrees with the results obtained by Navarro-Sempere et al. [61] in which they reported accumulations of heavy metals, such as mercury, in different areas of the brain, supporting the premise that the aetiology of mercury toxicity in the brain is the breakdown of the blood-brain barrier.

On the other hand, according to our previous research data [62] regarding the long-term exposure of animals to volcanic contaminants, not only the microglial cells have undergone changes but also differences in astrocytes were recorded between the studied populations: Mice from Furnas showed reactive astrogliosis, marked by an increase in GFAP (glial fibrillary acidic protein) and morphological transformation, as well as astrocyte dysfunction, with lower expression of the enzyme glutamine synthetase, when compared to individuals from Rabo de Peixe. Such events already indicated a possible proinflammatory response of the CNS to exposure to volcanic pollutants.

In this context, our previous studies and the present work have provided evidence for the existence of different inflammatory events in the brains of mice living in active volcanic environments, raising awareness about possible neurological health hazards in individuals inhabiting volcanically active areas. However, it should be noted that this neuroinflammatory process may not have a detrimental effect, as neuroinflammation may be playing a beneficial role.

## Data Availability

The data used to support the findings of this study can be found in the manuscript itself.

## References

[1] Fetler L., Amigorena S. (2005). NEUROSCIENCE: Brain under surveillance: the microglia patrol. *Science*.

[2] Li B., Dasgupta C., Huang L., Meng X., Zhang L. (2020). MiRNA-210 induces microglial activation and regulates microglia-mediated neuroinflammation in neonatal hypoxic-ischemic encephalopathy. *Cellular & Molecular Immunology*.

[3] Sierra A., de Castro F., del Río-Hortega J., Rafael Iglesias-Rozas J., Garrosa M., Kettenmann H. (2016). The “Big-Bang” for modern glial biology: translation and comments on Pío Del Río-Hortega 1919 series of papers on microglia. *Glia*.

[4] Lawson L. J., Perry V. H., Dri P., Gordon S. (1990). Heterogeneity in the distribution and morphology of microglia in the normal adult mouse brain. *Neuroscience*.

[5] Kettenmann H., Hanisch U.-K., Noda M., Verkhratsky A. (2011). Physiology of microglia. *Physiological Reviews*.

[6] Rock R. B., Gekker G., Hu S. (2004). Role of microglia in central nervous system infections. *Clinical Microbiology Reviews*.

[7] Cho B. P., Song D. Y., Sugama S. (2006). Pathological dynamics of activated microglia following medial forebrain bundle transection. *Glia*.

[8] Korzhevskii D. E., Kirik O. V. (2016). Brain microglia and microglial markers. *Neuroscience and Behavioral Physiology*.

[9] Parwaresch M. R., Radzun H. J., Kreipe H., Hansmann M. L., Barth J. (1986). Monocyte/macrophage-reactive monoclonal antibody Ki-M6 recognizes an intracytoplasmic antigen. *The American Journal of Pathology*.

[10] Smith M. J., Koch G. L. E. (1987). Differential expression of murine macrophage surface glycoprotein antigens in intracellular membranes. *Journal of Cell Science*.

[11] Nakajima K., Kohsaka S. (2001). Microglia: activation and their significance in the central nervous system. *Journal of Biochemistry*.

[12] Brown G. C., Vilalta A. (2015). How microglia kill neurons. *Brain Research*.

[13] Norris G. T., Kipnis J. (2019). Immune cells and CNS physiology: microglia and beyond. *The Journal of Experimental Medicine*.

[14] Marinelli S., Basilico B., Marrone M. C., Ragozzino D. (2019). Microglia-neuron crosstalk: signaling mechanism and control of synaptic transmission. *Seminars in Cell & Developmental Biology*.

[15] Vainchtein I. D., Molofsky A. V. (2020). Astrocytes and microglia: In sickness and in health. *Trends in Neurosciences*.

[16] Sivangala R., Sumanlatha G. (2015). Cytokines that mediate and regulate immune responses. *Innovative Immunology*.

[17] Montgomery S. L., Bowers W. J. (2012). Tumor necrosis factor-alpha and the roles it plays in homeostatic and degenerative processes within the central nervous system. *Journal of Neuroimmune Pharmacology*.

[18] Beattie E. C., Stellwagen D., Morishita W. (2002). Control of synaptic strength by glial TNFalpha. *Science*.

[19] Kaneko M., Stellwagen D., Malenka R. C., Stryker M. P. (2008). Tumor Necrosis Factor-*α* Mediates One Component of Competitive, Experience- Dependent Plasticity in Developing Visual Cortex. *Neuron*.

[20] Baune B. T., Wiede F., Braun A., Golledge J., Arolt V., Koerner H. (2008). Cognitive dysfunction in mice deficient for TNF- and its receptors. *American Journal of Medical Genetics Part B: Neuropsychiatric Genetics*.

[21] Beste C., Baune B. T., Falkenstein M., Konrad C. (2010). Variations in the TNF-*α* gene (TNF-*α* -308G→A) affect attention and action selection mechanisms in a dissociated fashion. *Journal of Neurophysiology*.

[22] Olmos G., Lladó J. (2014). Tumor necrosis factor alpha: a link between neuroinflammation and excitotoxicity. *Mediators of Inflammation*.

[23] Lyman M., Lloyd D. G., Ji X., Vizcaychipi M. P., Ma D. (2014). Neuroinflammation: the role and consequences. *Neuroscience Research*.

[24] Bazan N. G., Halabi A., Ertel M., Petasis N. A., Scott B., Siegel G. J., Albers R. W., Price D. L. (2012). Neuroinflammation. *Basic Neurochemistry*.

[25] Cheng S., Hou J., Zhang C. (2015). Minocycline reduces neuroinflammation but does not ameliorate neuron loss in a mouse model of neurodegeneration. *Scientific Reports*.

[26] Kirkley K. S., Popichak K. A., Hammond S. L., Davies C., Hunt L., Tjalkens R. B. (2019). Genetic suppression of IKK2/NF-*κ*B in astrocytes inhibits neuroinflammation and reduces neuronal loss in the MPTP-Probenecid model of Parkinson’s disease. *Neurobiology of Disease*.

[27] Wang Q., Oyarzabal E. A., Song S., Wilson B., Santos J. H., Hong J. S. (2020). Locus coeruleus neurons are most sensitive to chronic neuroinflammation-induced neurodegeneration. *Brain, Behavior, and Immunity*.

[28] Ransohoff R. M. (2016). How neuroinflammation contributes to neurodegeneration. *Science*.

[29] Heneka M. T., Carson M. J., Khoury J. (2015). Neuroinflammation in Alzheimer's disease. *The Lancet Neurology*.

[30] Calsolaro V., Edison P. (2016). Neuroinflammation in Alzheimer’s disease: current evidence and future directions. *Alzheimer's & Dementia*.

[31] Nazem A., Sankowski R., Bacher M., Al-Abed Y. (2015). Rodent models of neuroinflammation for Alzheimer’s disease. *Journal of Neuroinflammation*.

[32] Ardura-Fabregat A., Boddeke E. W. G. M., Boza-Serrano A. (2017). Targeting neuroinflammation to treat Alzheimer’s disease. *CNS Drugs*.

[33] Seung-Hoon Y. (2019). Cellular and molecular mediators of neuroinflammation in Alzheimer disease. *International Neurourology Journal*.

[34] Hampel H., Caraci F., Cuello A. C. (2020). A path toward precision medicine for neuroinflammatory mechanisms in Alzheimer’s disease. *Frontiers in Immunology*.

[35] Vivekanantham S., Shah S., Dewji R., Dewji A., Khatri C., Ologunde R. (2015). Neuroinflammation in Parkinson’s disease: role in neurodegeneration and tissue repair. *The International Journal of Neuroscience*.

[36] Gelders G., Baekelandt V., Van der Perren A. (2018). Linking neuroinflammation and neurodegeneration in Parkinson’s disease. *Journal of Immunology Research*.

[37] Liu Z., Qiu A. W., Huang Y. (2019). IL-17A exacerbates neuroinflammation and neurodegeneration by activating microglia in rodent models of Parkinson's disease. *Brain, Behavior, and Immunity*.

[38] Pegoretti V., Baron W., Laman J. D., Eisel U. L. M. (2018). Selective modulation of TNF-TNFRs signaling: insights for multiple sclerosis treatment. *Frontiers in Immunology*.

[39] Ribeiro C. M., Oliveira S. R., Alfieri D. F. (2019). Tumor necrosis factor alpha (TNF-*α*) and its soluble receptors are associated with disability, disability progression and clinical forms of multiple sclerosis. *Inflammation Research*.

[40] Wagner C. A., Roqué P. J., Goverman J. M. (2020). Pathogenic T cell cytokines in multiple sclerosis. *The Journal of Experimental Medicine*.

[41] El-Salem K., Al-Mistarehi A. H., Khalil H., Al-Sharman A., Yassin A. (2021). Serum tumor necrosis factor-alpha levels correlate with cognitive function scales scores in multiple sclerosis patients. *Multiple Sclerosis and Related Disorders*.

[42] Moulton P. V., Yang W. (2012). Air Pollution, Oxidative Stress, and Alzheimer's Disease. *Journal of Environmental and Public Health*.

[43] Calderón-Garcidueñas L., Solt A. C., Henríquez-Roldán C. (2008). Long-term air pollution exposure is associated with neuroinflammation, an altered innate immune response, disruption of the blood-brain barrier, ultrafine particulate deposition, and accumulation of amyloid *β*-42 and *α*-synuclein in children and young adults. *Toxicologic Pathology*.

[44] Wang J., Ma T., Ma D. (2021). The impact of air pollution on neurodegenerative diseases. *Therapeutic Drug Monitoring*.

[45] Block M. L., Calderón-Garcidueñas L. (2009). Air pollution: mechanisms of neuroinflammation and CNS disease. *Trends in Neurosciences*.

[46] Calderón-Garcidueñas L., Azzarelli B., Acuna H. (2002). Air pollution and brain damage. *Toxicologic Pathology*.

[47] Levesque S., Surace M. J., McDonald J., Block M. L. (2011). Air pollution and the brain: subchronic diesel exhaust exposure causes neuroinflammation and elevates early markers of neurodegenerative disease. *Journal of Neuroinflammation*.

[48] Jayaraj R. L., Rodriguez E. A., Wang Y., Block M. L. (2017). Outdoor ambient air pollution and neurodegenerative diseases: the neuroinflammation hypothesis. *Current Environmental Health Reports*.

[49] Calderón-Garcidueñas L., Leray E., Heydarpour P., Torres-Jardón R., Reis J. (2016). Air pollution, a rising environmental risk factor for cognition, neuroinflammation and neurodegeneration: the clinical impact on children and beyond. *Revue Neurologique*.

[50] Calderón-Garcidueñas L., Kavanaugh M., Block M. (2012). Neuroinflammation, hyperphosphorylated tau, diffuse amyloid plaques, and down-regulation of the cellular prion protein in air pollution exposed children and young adults. *Journal of Alzheimer's Disease*.

[51] Peters R., Ee N., Peters J., Booth A., Mudway I., Anstey K. J. (2019). Air pollution and dementia: a systematic review. *Journal of Alzheimer's Disease*.

[52] Costa L. G., Cole T. B., Dao K., Chang Y. C., Coburn J., Garrick J. M. (2020). Effects of air pollution on the nervous system and its possible role in neurodevelopmental and neurodegenerative disorders. *Pharmacology & Therapeutics*.

[53] Kelman I., Mather T. A. (2008). Living with volcanoes: The sustainable livelihoods approach for volcano-related opportunities. *Journal of Volcanology and Geothermal Research*.

[54] Sigurdsson H., Houghton B., McNutt S., Rymer H., Stix J. (2015). *Encyclopedia of Volcanoes*.

[55] Linhares D., Garcia P. V., Viveiros F., Ferreira T., Rodrigues A. . S. (2015). Air pollution by hydrothermal volcanism and human pulmonary function. *BioMed Research International*.

[56] Hansell A., Oppenheimer C. (2004). Health hazards from volcanic gases: a systematic literature review. *Archives of Environmental Health*.

[57] Holmberg K., Small C. (2015). People and volcanoes: how many and how Closc?. *Fall Meeting of the American Geophysical Union*.

[58] Viveiros F., Cardellini C., Ferreira T., Caliro S., Chiodini G., Silva C. (2010). Soil CO2 emissions at Furnas volcano, São Miguel Island, Azores archipelago: volcano monitoring perspectives, geomorphologic studies, and land use planning application.

[59] Amaral A., Cabral C., Guedes C., Rodrigues A. (2007). Apoptosis, metallothionein, and bioavailable metals in domestic mice (Mus musculus L.) from a human-inhabited volcanic area. *Ecotoxicology*.

[60] Amaral A., Rodrigues V., Oliveira J. (2006). Chronic exposure to volcanic environments and cancer incidence in the Azores. *Science of The Total Environment*.

[61] Navarro-Sempere A., Segovia Y., Rodrigues A. S., Garcia P. V., Camarinho R., García M. (2021). First record on mercury accumulation in mice brain living in active volcanic environments: a cytochemical approach. *Environmental Geochemistry and Health*.

[62] Navarro A., García M., Rodrigues A. S., Garcia P. V., Camarinho R., Segovia Y. (2021). Reactive astrogliosis in the dentate gyrus of mice exposed to active volcanic environments. *Journal of Toxicology and Environmental Health, Part A*.

[63] Bagnato E., Barra M., Cardellini C., Chiodini G., Parello F., Sprovieri M. (2014). First combined flux chamber survey of mercury and CO2 emissions from soil diffuse degassing at Solfatara of Pozzuoli crater, Campi Flegrei (Italy): mapping and quantification of gas release. *Journal of Volcanology and Geothermal Research*.

[64] Bagnato E., Viveiros F., Pacheco J. E., D’Agostino F., Silva C., Zanon V. (2018). Hg and CO2 emissions from soil diffuse degassing and fumaroles at Furnas volcano (São Miguel Island, Azores): gas flux and thermal energy output. *Journal of Geochemical Exploration*.

[65] Durand M., Florkowski C., George P., Walmsley T., Weinstein P., Cole J. (2004). Elevated trace element output in urine following acute volcanic gas exposure. *Journal of Volcanology and Geothermal Research*.

[66] Forsyth D., Richard F., Rattner B. (2001). Extrapolation of laboratory tests to field populationsitle. *Ecotoxicology of wild mammals; Shore*.

[67] Tersago K., de Coen W., Scheirs J. (2004). Immunotoxicology in wood mice along a heavy metal pollution gradient. *Environmental Pollution*.

[68] Quere J., Vincent J. (1989). Determination de l’âge chez le mulot gris (Apodemus sylvaticus L., 1758) par la pesee des cristallins. *Mammalia*.

[69] Zotova E., Bharambe V., Cheaveau M. (2013). Inflammatory components in human Alzheimer’s disease and after active amyloid-*Β*_42_ immunization. *Brain*.

[70] Linhares D., Garcia P., Rodrigues A., Murillo-Tovar M., Saldarriaga-Noreña H., Saeid A. (2020). Trace elements in volcanic environments and human health effects. *Trace Elements in the Environment-New Approaches and Recent Advances*.

[71] Camarinho R., Garcia P. V., Choi H., Rodrigues A. S. (2019). Overproduction of TNF-*Α* and lung structural remodelling due to chronic exposure to volcanogenic air pollution. *Chemosphere*.

[72] Linhares D. P. S., Garcia P. V., Silva C. (2018). DNA damage in oral epithelial cells of individuals chronically exposed to indoor radon (222Rn) in a hydrothermal area. *Environmental Geochemistry and Health*.

[73] Ferreira A. F., Garcia P. V., Camarinho R., dos Santos Rodrigues A. (2015). Volcanogenic pollution and testicular damage in wild mice. *Chemosphere*.

[74] Camarinho R., Garcia P. V., Rodrigues A. S. (2013). Chronic exposure to volcanogenic air pollution as cause of lung injury. *Environmental Pollution*.

[75] Brockhaus J., Möller T., Kettenmann H. (1996). Phagocytozing ameboid microglial cells studied in a mouse corpus callosum slice preparation. *Glia*.

[76] Petersen M. A., Dailey M. E. (2004). Diverse microglial motility behaviors during clearance of dead cells in hippocampal slices. *Glia*.

[77] Kölliker-frers R., Udovin L., Otero-losada M. (2021). Neuroinflammation: An Integrating Overview of Reactive-Neuroimmune Cell Interactions in Health and Disease. *Mediators of Inflammation*.

[78] Li T., Zhang S. (2016). Microgliosis in the injured Brain. *The Neuroscientist*.

[79] Uysal N., Tugyan K., Aksu I. (2012). Age-related changes in apoptosis in rat hippocampus induced by oxidative stress. *Biotechnic & Histochemistry*.

[80] Eriksson P. S., Perfilieva E., Björk-Eriksson T. (1998). Neurogenesis in the adult human hippocampus. *Nature Medicine*.

[81] Lieberman A. P., Pitha P. M., Shin H. S., Shin M. L. (1989). Production of tumor necrosis factor and other cytokines by astrocytes stimulated with lipopolysaccharide or a neurotropic virus. *Proceedings of the National Academy of Sciences of the United States of America*.

[82] Liu X. S., Luo Z. H., Yang Z. C., Huang W. H., Li A. (1994). The significance of changes in serum tumour necrosis factor (TNF) activity in severely burned patients. *Burns*.

[83] Morganti-Kossman M. C., Lenzlinger P. M., Hans V. (1997). Production of cytokines following brain injury: beneficial and deleterious for the damaged tissue. *Molecular Psychiatry*.

[84] Lau L. T., Yu A. C. H. (2001). Astrocytes produce and release interleukin-1, interleukin-6, tumor necrosis factor alpha and interferon-gamma following traumatic and metabolic injury. *Journal of Neurotrauma*.

[85] Hanisch U. K. (2002). Microglia as a source and target of cytokines. *Glia*.

[86] Klintworth H., Garden G., Xia Z. (2009). Rotenone and paraquat do not directly activate microglia or induce inflammatory cytokine release. *Neuroscience Letters*.

[87] Baumann B., Seufert J., Jakob F. (2005). Activation of NF-*Κ*B signalling and TNF*α*-expression in THP-1 macrophages by TiAlV- and polyethylene-wear particles. *Journal of Orthopaedic Research*.

[88] Ben-Neriah Y., Karin M. (2011). Inflammation meets cancer, with NF-*κ*B as the matchmaker. *Nature Immunology*.

[89] Smale S. T. (2011). Hierarchies of NF-*κ*B target-gene regulation. *Nature Immunology*.

[90] Hayden M. S., Ghosh S. (2012). NF-*κ*B, the first quarter-century: remarkable progress and outstanding questions. *Genes & Development*.

[91] Widera D., Mikenberg I., Kaltschmidt B., Kaltschmidt C. (2006). Potential role of NF-*κ*B in adult neural stem cells: the underrated steersman?. *International Journal of Developmental Neuroscience*.

[92] Pluchino S., Zanotti L., Rossi B. (2005). Neurosphere-derived multipotent precursors promote neuroprotection by an immunomodulatory mechanism. *Nature*.

[93] Gao H. M., Hong J. S. (2008). Why neurodegenerative diseases are progressive: uncontrolled inflammation drives disease progression. *Trends in Immunology*.

[94] Stolp H. B., Dziegielewska K. M. (2009). Review: role of developmental inflammation and blood-brain barrier dysfunction in neurodevelopmental and neurodegenerative diseases. *Neuropathology and Applied Neurobiology*.

[95] Kumar A. (2018). Editorial: Neuroinflammation and cognition. *Frontiers in Aging Neuroscience*.

[96] Kohman R. A., Rhodes J. S. (2013). Neurogenesis, inflammation and behavior. *Brain, Behavior, and Immunity*.

[97] Yirmiya R., Goshen I. (2011). Immune modulation of learning, memory, neural plasticity and neurogenesis. *Brain, Behavior, and Immunity*.

[98] Cole T. B., Coburn J., Dao K. (2016). Sex and genetic differences in the effects of acute diesel exhaust exposure on inflammation and oxidative stress in mouse brain. *Toxicology*.

[99] Bai K. J., Chuang K. J., Chen C. L. (2019). Microglial activation and inflammation caused by traffic-related particulate matter. *Chemico-Biological Interactions*.

[100] Cheng H., Davis D. A., Hasheminassab S., Sioutas C., Morgan T. E., Finch C. E. (2016). Urban traffic-derived nanoparticulate matter reduces neurite outgrowth via TNF*α* in vitro. *Journal of Neuroinflammation*.

[101] Morgan T. E., Davis D. A., Iwata N. (2011). Glutamatergic neurons in rodent models respond to nanoscale particulate urban air pollutants in vivo and in vitro. *Environmental Health Perspectives*.

[102] Luo C., Koyama R., Ikegaya Y. (2016). Microglia engulf viable newborn cells in the epileptic dentate gyrus. *Glia*.

